# Dental manifestations in adult hypophosphatasia and their correlation with biomarkers

**DOI:** 10.1002/jmd2.12307

**Published:** 2022-06-28

**Authors:** Priya Sinha, Rachel Gabor, Rachael Haupt‐Harrington, Leila Deering, Robert D. Steiner

**Affiliations:** ^1^ Marshfield Clinic Health System Marshfield Dental Center Marshfield Wisconsin USA; ^2^ Marshfield Clinic Research Institute Research Computing and Analytics Marshfield Wisconsin USA; ^3^ Marshfield Clinic Health System, Marshfield Clinic Research Institute Medical Genetics Marshfield Wisconsin; ^4^ University of Wisconsin School of Medicine and Public Health Madison Wisconsin

**Keywords:** alkaline phosphatase, asfotase alfa, dental, enzyme replacement therapy, hypophosphatasia, periodontal breakdown, tooth loss

## Abstract

Hypophosphatasia (HPP) is a genetic condition with broad clinical manifestations caused by alkaline phosphatase (ALP) deficiency. Adults with HPP exhibit a wide spectrum of signs and symptoms. Dental manifestations including premature tooth loss are common. Much of the published literature reporting dental manifestations consists of case reports and series of symptomatic patients, likely biased towards more severe dental manifestations. The objective of this study was to systematically explore the dental manifestations among adults with HPP by conducting a comprehensive dental evaluation. To minimize bias, the study explored dental manifestations in an unselected cohort of adults with HPP. Participants were identified searching electronic health record (EHR) data from a rural health system to discover adults with persistent ALP deficiency. Heterozygotes with pathogenic (P), likely pathogenic (LP), or uncertain variants (VUS) in *ALPL* and at least one elevated ALP substrate were defined as adults with HPP and underwent genetic, dental, oral radiographic, and biomarker evaluation. Twenty‐seven participants completed the study. Premature tooth loss was present in 63% (17/27); 19% (5/27) were missing eight or more teeth. Statistically significant associations were found between premature permanent tooth loss and HPP biomarkers ALP (*p* = 0.049) and bone‐specific ALP (*p* = 0.006). Serum ALP (*ρ* = −0.43, *p* = 0.037) and bone‐specific ALP (*ρ* = −0.57, *p* = 0.004) were negatively correlated with number of teeth lost prematurely. As noted with tooth loss, periodontal breakdown was associated with bone‐specific ALP. An inverse association between periodontal breakdown and bone‐specific ALP was observed (*p* = 0.014). These findings suggest a role for ALP in maintenance of dentition.

Synopsis

Adults with hypophosphatasia identified by electronic health records search exhibit significant dental manifestations which are associated with alkaline phosphatase activity, suggesting a role for the enzyme in maintenance of dentition.

## 1 INTRODUCTION

Hypophosphatasia (HPP) is a rare, inherited mineralization disorder involving primarily bones and teeth. Affected individuals have deficient alkaline phosphatase (ALP) and pathogenic variant(s) in *ALPL*, the gene encoding the tissue non‐specific alkaline phosphatase (TNSALP) enzyme.^1^, ^2^ Phosphatase activity of TNSALP (hereafter referred to as ALP) is required for normal mineralization, essentially the deposition of calcium and phosphorus in developing bones and teeth, consequently, the enzyme is critical for the resultant strength and durability of the skeleton and dentition. Persistently low ALP activity results in accumulation of ALP substrates including pyridoxal phosphate (PLP), phosphoethanolamine (PEA), and inorganic pyrophosphate (PPi) typically measured in plasma, urine and extracellular space, respectively, the former two available as routine clinical tests and serving as disease biomarkers.^1^, ^2^, ^3^ Accumulated extracellular PPi impairs mineralization of hard tissues, and is thought to result in defective mineralization responsible for the manifestations of HPP.

Several clinical forms of HPP are described including perinatal, infantile, childhood, adult, and odontohypophosphatasia (odonto‐HPP), representing phenotypes ranging from profoundly severe to very mild.^1^, ^2^, ^4^ At its most severe, HPP results in a stillborn infant essentially lacking bone mineralization; at its mildest, HPP is limited to early tooth loss. Across the life‐span, varying degrees of rickets, osteomalacia, and premature tooth loss are noted with varying frequency and severity. The genetics of HPP are complex, the severe forms usually demonstrating autosomal recessive inheritance (with biallelic *ALPL* variants), while mild forms are often autosomal dominant. Adults with HPP typically demonstrate osteomalacic pseudo‐fractures and stress fractures sometimes accompanied by early loss of permanent dentition along with other variable features.^4^, ^5^ Dental manifestations are poorly characterized among adults with HPP.^6^ It is also documented that patients with isolated dental manifestation of HPP (odontohypophosphatasia) in childhood may later develop additional manifestations, justifying attempts at reclassification.^7^

Only limited literature exists on the prevalence of adults with HPP due to the heterogeneity of clinical manifestations and the lower rate of diagnosis.^5^, ^8^ The combined prevalence of mild to moderate forms of HPP is estimated at one in 6300 among Europeans.^6^ Recent findings from a large cohort study also reported that the prevalence of mild to moderate forms of HPP is higher than expected and often underestimated.^9^ Furthermore, it is likely that a majority of adults with HPP encounter delayed diagnosis, misdiagnosis or remain undiagnosed because individuals with HPP are occasionally asymptomatic when identified fortuitously or exhibit very mild, non‐specific symptoms.^10^, ^11^, ^12^, ^13^ Berkseth et al. found 9% of patients with adult HPP with a history of childhood rickets not diagnosed with HPP as children.^14^ Weber et al documented that half of a cohort of HPP patients reported childhood onset of symptoms.^15^ A previous study in the same large, rural multispecialty clinic in which this current study was conducted estimated the prevalence of persistent hypophosphatasemia (serum alkaline phosphatase <30 IU/L) at one in 1544 adults and speculated that a subset of these individuals may fall within the spectrum of HPP.^11^

Severe forms are more frequently observed in a clinical setting due to observable clinical manifestations.^16^ Untreated patients with severe perinatal or infantile HPP have high morbidity and mortality.^4^ Treatment with asfotase alfa, a bone‐targeted alkaline phosphatase enzyme replacement therapy (ERT), is marketed for pediatric patients with perinatal/infantile‐ and juvenile‐onset HPP.^4^ Among adults with HPP a significant reduction in plasma concentrations of PLP to levels within normal ranges by 6 months of asfotase alfa treatment has been documented. In addition, significant decrease in mineralization lag time was also observed following 1 year of therapy.^17^ Recently, Okawa et al published a case report of deciduous teeth erupting following initiation of ERT and stable periodontal conditions the authors postulated was due to improvement in alveolar bone and tooth mineralization. Their findings also suggested that ERT is not expected to recover periodontal conditions of erupted teeth.^18^ Schroth et al reported that infants receiving ERT lost significantly fewer teeth prematurely to HPP than those starting ERT at a later age.^19^

There is a paucity of published information on the dental manifestations of HPP in milder forms, and factors affecting the severity of dental involvement are poorly understood. Dental findings in HPP have been more commonly reported and with greater detail in primary (deciduous) dentition and among individuals with childhood HPP where premature exfoliation of deciduous teeth has been documented.^20^ Adults with HPP often report a history of premature deciduous tooth loss in childhood. The description of dental manifestations among adults with HPP in the literature is limited to individual case reports, family studies, or small case series, which primarily illustrate relatively severe presentations; there is likely a bias towards reporting more severe dental manifestations, since case reports and series generally report symptomatic patients coming to attention due to dental problems. Our review of the literature did not identify any descriptions of dental manifestations in patient cohorts ascertained in an unbiased manner by ALP measurement rather than signs or symptoms. Few studies have also reported early loss of permanent dentition and/or increased tooth mobility.^5^, ^14^, ^21^ Premature tooth loss is common in HPP, but the etiology is unclear. Tooth mobility and subsequent loss may be due to secondary loss of alveolar bone support.^3^, ^20^ It has been suggested that pathogenic variants in *ALPL* can cause several alterations in alveolar bone properties including defective or delay in bone mineralization and resorptive lesions thereby increasing the risk for periodontal disease and early loss of teeth due to poor attachment of tooth roots to bone via periodontal ligament.^22^ Additional dental findings such as tooth enamel hypoplasia or hypomineralization, dental caries, enlarged pulp chambers, and short roots have also been described in permanent dentition among adults with HPP.^20^ Enamel hypoplasia may lead to occlusal problems (e.g., tooth wear and cracked tooth), increase susceptibility to dental caries, and early pulpal involvement. Okawa et al reported that spontaneous early loss of deciduous teeth was more commonly found in odonto‐HPP; conversely tooth hypo mineralization, malocclusion, and permanent tooth mobility predominantly occurred in non‐odonto HPP.^23^ Clearly, there are many unanswered questions related to dental manifestations in HPP, especially in milder forms.

The focus of this work is HPP in adults, especially their dentition and the association of tooth loss with disease biomarkers to hopefully begin to shed light on pathogenesis. We did not define the population as adult HPP, because we did not exclude individuals with earlier onset of signs/symptoms; rather, we defined the population as adults with HPP.

## 2 METHODS

### 2.1 Study cohort

This study was supported by an investigator‐initiated research grant (Investigator Sponsored Trial) from Alexion Pharmaceuticals and approved by the Marshfield Clinic Institutional Review Board. Study data were collected over approximately 2 years with the first patient visit in the study in September 2018 and the last visit in the study taking place in October 2019. All participants consented to enrollment in the study.

**FIGURE 1:**
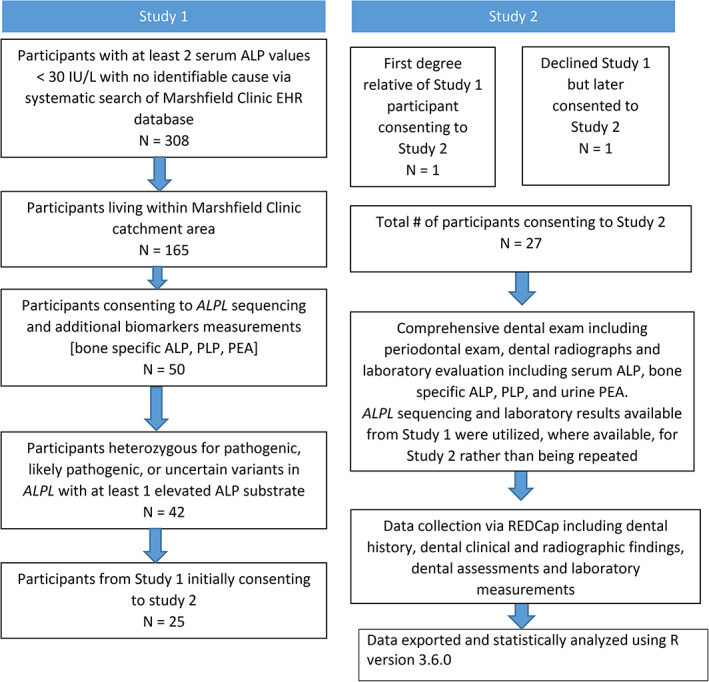
Study design. Design of the study illustrating ascertainment, recruitment, and enrollment of participants, study procedures, and relationship between the previously published study (Study 1) and the current study (Study 2). Also shown are data collection and statistical methods employed in the study

The present study followed an earlier study in the same large multispecialty rural health system designed to ascertain the prevalence and clinical characteristics of adults with hypophosphatasemia in a large unbiased sample and is an extension of that earlier study (Figure 1, Study 1).^5^ Our former colleague Fergus McKiernan and collaborators identified a cohort of individuals with low ALP levels who might have HPP via a systematic search of the health system EHR database. Individuals with low ALP were then tested for variants in *ALPL*, and those with such P, LP, or VUS variants were invited to participate in further investigation. The purpose of the current study (Figure 1, Study 2) was to explore the dental phenotype among adults with HPP defined by low ALP and *ALPL* pathogenic variants or VUS in *ALPL* with at least one elevated ALP substrate by conducting a comprehensive dental examination and full mouth radiographs. Full clinical evaluation, genetic and pedigree analysis, quality of life surveys, and skeletal radiographs, as well as additional laboratory testing were all carried out; however, the findings of those evaluations are beyond the scope of this manuscript, and will be reported separately.

The earlier study from which this study built upon identified 308 patients, 18 years‐of‐age or older, with at least two serum ALP values <30 IU/L, measured between January 1, 2002 and January 1, 2015, for which no other cause was readily identified.^10^ Within this pool, 165 individuals found to be living within the Marshfield Clinic catchment area were invited to participate in the research. Of those, 50 who consented to the earlier study agreed to have *ALPL* genetic testing and ALP biomarker measurements. Of the 50 participants, 42 were heterozygous for pathogenic, likely pathogenic, or uncertain variants in *ALPL*. Those 42 participants were invited to participate in the current study. HPP biomarkers in this study are defined as ALP, bone‐specific ALP, PLP, and PEA. None of the participants had previously been diagnosed with HPP; 25 participated, plus one first degree relative of an initial participant, and one participant who had declined participation in the earlier study but agreed to be screened for the current study and qualified based on genetic and biomarker results. A total of 27 individuals participated in the current observational study; all 27 completed the study. Participants acknowledged potential risks and benefits and consented to participate.

### 2.2 Dental assessment

Personal and family dental history was obtained, full mouth radiographs were taken and interpreted and a comprehensive dental examination including periodontal findings were completed. Laboratory evaluation included measurement of the following HPP biomarkers: serum ALP, bone‐specific ALP, plasma PLP, spot urine PEA (plasma PLP and urine PEA data were extracted from the earlier study data, except for two new participants who did not complete the earlier study and underwent prospective testing). *ALPL* sequencing was performed (PreventionGenetics, Marshfield, WI). Biochemical measurements were performed at Marshfield Clinic Labs except for urine PEA (Mayo Laboratories, Rochester, MN). Investigators queried whether participants were receiving or had received ERT; only a single participant was receiving ERT prior to and during the course of the study. ERT was begun approximately 1 year prior to enrollment in the study at age 50 years and continued throughout the duration of the study and beyond. That individual's alkaline phosphatase data included in the study was taken from medical records prior to ERT administration as noted in the results section.

### 2.3 Data collection and statistical analysis

A standard REDCap (Research Electronic Data Capture) database was created for data collection.^24^ REDCap is a secure, web‐based software platform designed to support data capture for research studies, providing (1) an intuitive interface for validated data capture; (2) audit trails for tracking data manipulation and export procedures; (3) automated export procedures for seamless data downloads to common statistical packages; and (4) procedures for data integration and interoperability with external sources. Study data were collected and managed using REDCap electronic data capture tools hosted at Marshfield Clinic Research Institute. REDCap's repeatable instruments were used to collect dental history; dental clinical and radiographic findings and assessments; and genetic and biomarker testing. Once data collection was complete, the data were exported and statistically analyzed.

Premature tooth loss in deciduous dentition is defined as tooth loss at <5 years‐of‐age. Unlike with deciduous dentition, there is no clear definition of premature tooth loss for adult dentition. Multiple risk factors such as decay, periodontal disease, etc., cause loss of permanent teeth. Aging is also associated with tooth loss. Therefore, defining premature tooth loss in adults is challenging. Premature tooth loss was described herein by grouping tooth loss into categories of none, one tooth, two to eight teeth, and more than eight teeth, and the distribution of premature tooth loss among HPP participants was reported as counts and percentages. The statistical analysis of dental and biomarker evaluations was primarily descriptive, but inferential statistics were used when appropriate. All analyses were completed using R version 3.6.0.^25^ Concordance between periodontal status determined by radiographic examinations and by clinical evaluations was assessed using the Spearman correlation coefficient. Demographic characteristics, general and dental health measures, dental hygiene practices, and HPP biomarkers were also reported using counts and percentages for categorical variables and medians and interquartile ranges for continuous variables. Dental hygiene practices, dental health measures, and HPP biomarkers for participants who had any premature tooth loss and those who did not were compared using a Wilcoxon rank‐sum test or Fisher's exact test, as appropriate. All comparisons were two‐tailed, with *p* ≤ 0.05 considered significant.

The following clinical parameters were measured for all teeth (excluding third molars and retained deciduous teeth) to determine periodontal disease status (or periodontitis types): Periodontal pocket depths, bleeding on probing, gingival margin, clinical attachment loss, tooth mobility, and furcation. Bitewing radiographs, a subset of full mouth radiographs, were also used to assist in periodontal disease classification by measuring loss of alveolar bone height. Hausmann et al. concluded that a relationship exists between clinical attachment and radiographic bone height.^26^ Therefore, both clinical measures and dental radiographs were used to assess periodontal disease status (or severity) type. American Academy of Periodontology (AAP)/American Dental Association (ADA) periodontal classification system was used to categorize periodontal disease status.^27^ Participants found to be completely edentulous were allocated as periodontal status type VI for this study to better compare the severity of periodontal breakdown. The study also explored and documented presence or absence of dental defects including enamel hypoplasia, short roots, enlarged pulp chambers, or taurodontism among the participants.

The association of ALP level with periodontal disease status and dental caries were measured using Spearman's rank correlation and Wilcoxon rank sum tests respectively. For the purpose of this study, we separately defined current or historical periodontal breakdown from periodontal disease status. Current periodontal breakdown status was ascribed to all participants with periodontal disease type III, IV, and VI. Historical periodontal breakdown was ascribed to participants with current periodontitis type II who also had a history of premature tooth loss in an attempt to incorporate participants who may have previously lost teeth prematurely but currently maintain good periodontal health. Periodontal breakdown and the association with bone‐specific ALP level was assessed using Wilcoxon rank‐sum tests.

The association of ALP level with coronal dentin thickness and number of teeth lost prematurely was assessed using the Spearman correlation coefficient. We fit a Poisson model for number of teeth lost prematurely regressed on ALP level with patient age as a potential covariate and modifier. Models were evaluated using adjusted R^2^, AIC, and likelihood ratio tests for nested models only. The best fit model included ALP level and participant age greater than 65 (defined as elderly).

## 3 RESULTS

Participant age ranged from 40 to 86 with median age of 63 years. Gender predilection was found, with females accounting for 67% of participants. The gender difference is similar to those previously reported.^14^, ^15^ A majority of the participants (approximately 89%) reported good general health (Table 1). Overall, participants reported good dental hygiene practices; 67% reported brushing their teeth at least twice daily and 44% flossing at least daily. Approximately 70% of the participants had received their most recent dental examination within 1 year (Table 4). Nearly 26% reported having had treatment for periodontal disease (or deep cleaning) in the past, but a majority were unsure whether they had undergone these interventions prior to the start of the study. Of participants, 67% recalled having no early loss of deciduous teeth. A strong family history of early denture use was reported; 52% stated either their parents or first degree relatives used dentures prior to age 55.

**TABLE 1:** Demographic and general health characteristics for HPP participants

Gender	
Male	9 (33.3)
Female	18 (66.7)
Age, years	63.0 (20.0)
Age	
36–45 years	1 (3.7)
46–55 years	9 (33.3)
56–65 years	5 (18.5)
66–75 years	8 (29.6)
76–85 years	3 (11.1)
86 years and older	1 (3.7)
General health, self‐reported	
Excellent	4 (14.8)
Very good	8 (29.6)
Good	12 (44.4)
Fair	3 (11.1)
Ability to complete moderate activities	
Not limited at all	14 (51.9)
Limited a little	12 (44.4)
Limited a lot	1 (3.7)

*Note*: Medians and IQR are reported for continuous variable. Counts and percentages are reported for categorical variables.

**TABLE 2:** Median lab values and range for HPP participants

	Median	Range	Normal Range
ALP^a^ (IU/L)	22	5–36	40–125
Bone‐specific ALP^a^ (μg/L)	4	1–9	0–22
Pyridoxal phosphate (μg/L)	60	22–514	5–50
Urine phosphoethanolamine (nmol/mg/creat)	109	24–1024	<48

^a^ ALP and bone‐specific ALP results from historical testing were used for one participant who began treatment with asfotase alfa which can affect these levels. Results from historical testing for bone‐specific ALP lab was used for another participant who did not have this lab specifically completed for this study.

**TABLE 3:** Median biochemical measurements and range for HPP patients by genetic variant

	Pathogenic *N* = 21	Likely pathogenic *N* = 3	VUS *N* = 3
ALP (U/L)	22 (5–36)	24 (14–27)	17 (14–19)
Bone‐specific ALP^a^ (μg/dL)	4 (1–9)	6 (2–7)	3 (2–4)
Pyridoxal phosphate (μg/L)	60 (22–171)	208 (26–514)	52 (22–158)
Urine phosphoethanolamine (nmol/mg/creat)	82 (24–1024)	169 (64–622)	172 (47–180)

Abbreviations: ALP, alkaline phosphatase. VUS, variant of uncertain significance.

HPP biomarkers including ALP, bone‐specific ALP, PLP, and PEA were collected, compared, and analyzed for all participants (Tables 2, 3). In this cohort, not unexpectedly and in keeping with the inclusion criteria, we found universally low ALP levels, and predominantly high PLP and PEA levels among the participants.

**TABLE 4:** Dental hygiene and oral health characteristics in adult HPP in those with and without history of premature tooth loss

	No premature tooth loss *N* = 10	Premature tooth loss *N* = 17	*p*‐value
ALP	23.5 (12)	19.0 (9.0)	0.049
Bone‐specific ALP	5.5 (2.3)	3.0 (1.0)	0.006
Pyridoxal phosphate	68.5 (106.0)	60.0 (77.0)	0.514
Urine PEA	81.5 (45.8)	114.0 (130.0)	0.786
Periodontal Status/Severity			0.381
II	7 (70.0)	10 (58.8)	
III	3 (30.0)	3 (17.6)	
IV	0 (0)	2 (11.8)	
VI	0 (0)	2 (11.8)	
Time since last dental examination			0.244
Within last year	9 (90.0)	10 (58.8)	
Within 1–3 years	1 (10.0)	3 (17.6)	
More than 3 years	0 (0)	4 (23.5)	
Brushing frequency			0.500
More than twice daily	2 (20.0)	1 (5.9)	
Twice daily	5 (50.0)	10 (58.8)	
Once daily	2 (20.0)	4 (23.5)	
Less than once daily	1 (10.0)	0 (0)	
Unknown	0 (0)	2 (11.8)	
Flossing frequency			0.520
Twice daily	2 (20.0)	1 (5.9)	
Once daily	4 (40.0)	5 (29.4)	
Less than once daily	4 (40.0)	9 (52.9)	
Unknown	0 (0)	2 (11.8)	
Dental caries			0.221
No	8 (80.0)	8 (47.1)	
Yes	1 (10.0)	7 (41.2)	
Unknown	1 (10.0)	2 (11.8)	
Deep Cleaning			
Yes	1 (10.0)	6 (35.3)	0.204
Unsure	9 (90.0)	11 (64.3)	

*Note*: Medians, IQR and Wilcoxon rank sum test p‐values are reported for continuous variable. Counts, percentages, and Fisher exact test p‐values are reported for categorical variables. Counts, percentages, and Wilcoxon rank‐sum test p‐values are reported for ordinal data (periodontal status/severity).

Abbreviations: ALP, alkaline phosphatase; PEA, phosphoethanolamine.

**FIGURE 2:**
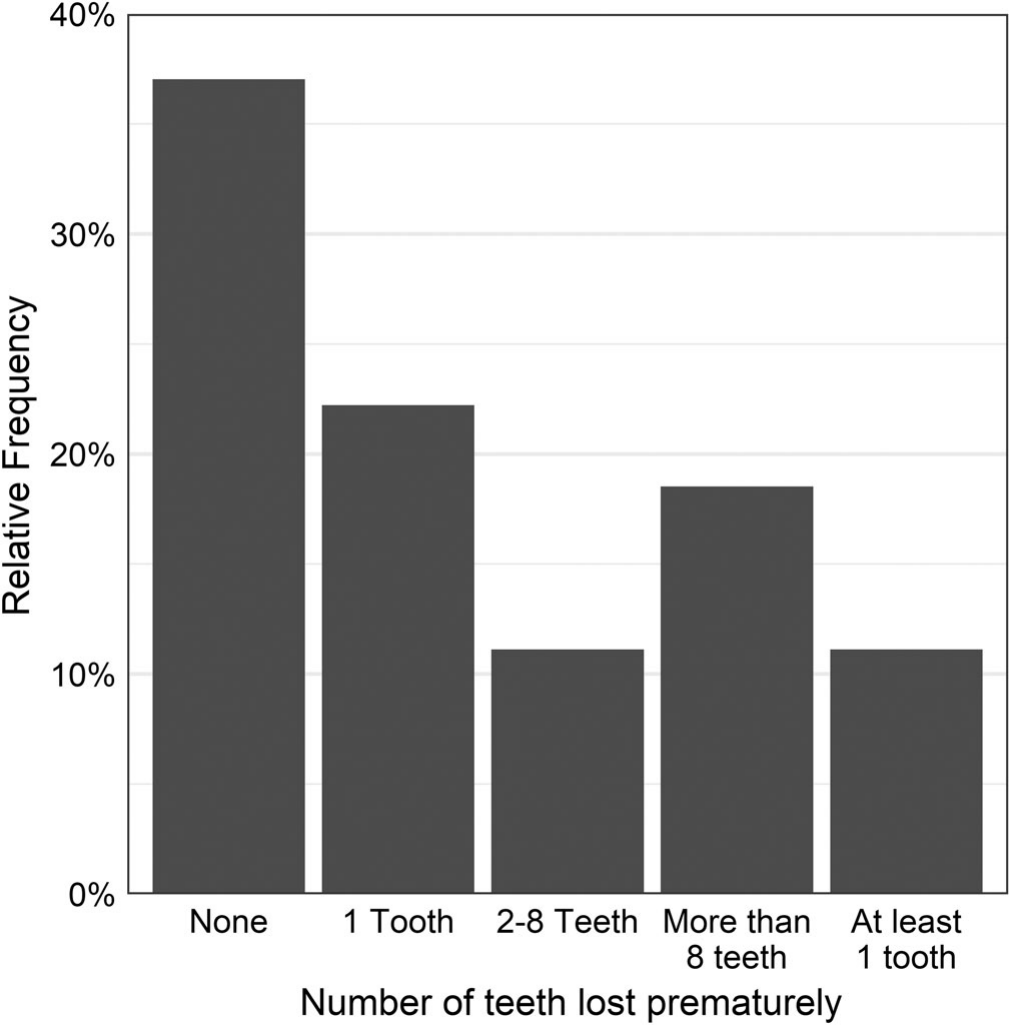
Relative frequency of number of teeth lost prematurely among HPP participants. Premature tooth loss was present in 63% (*N* = 17) of HPP patients. Three patients had premature tooth loss but an unknown number of teeth lost

**FIGURE 3:**
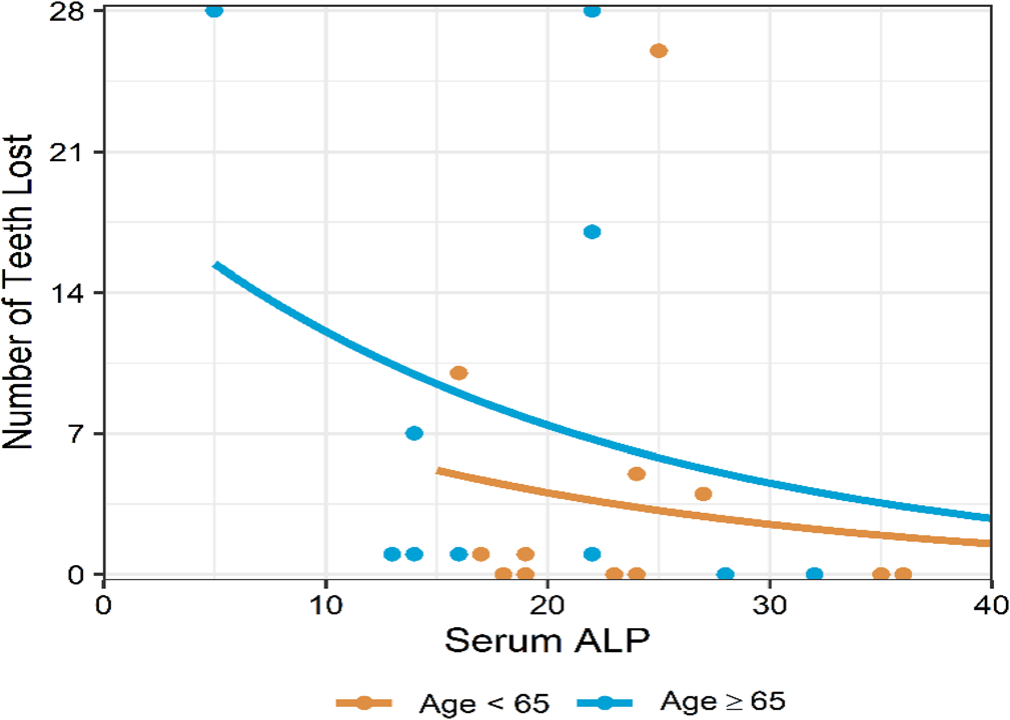
Serum ALP was negatively associated with number of teeth lost (Incidence ratio: 0.952, *p* < 0.001). Participant age was also associated with the number of teeth lost with participants aged 65 and older experiencing more teeth lost independent of serum ALP levels (*p* = 0.002). The incident rate of tooth loss among participants age 65 and older are 1.82 times the incident rate of younger participants. Estimates are from a Poisson model regressing number of teeth lost on serum ALP and age indicator. Excludes participants with premature tooth loss but unknown number (*N* = 3)

**FIGURE 4:**
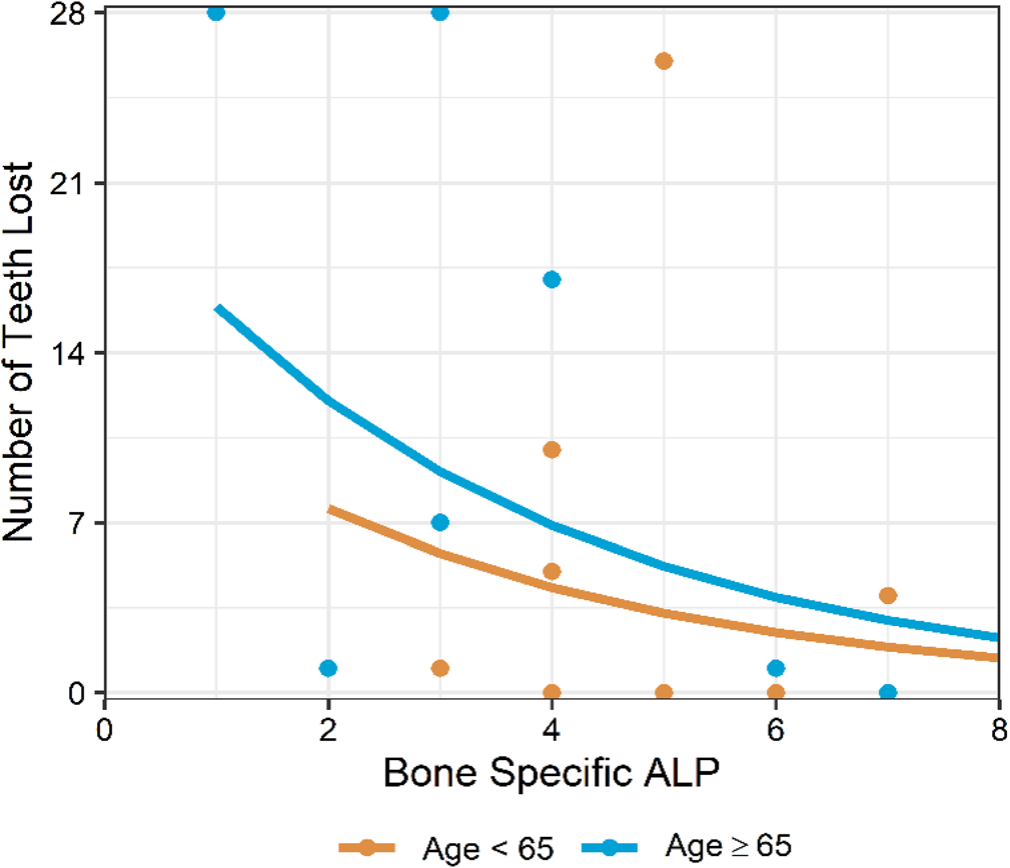
Bone‐specific ALP was negatively associated with number of teeth lost (Incidence ratio: 0.757, *p* < 0.001). Participant age was also associated with the number of teeth lost with participants aged 65 and older experiencing more teeth lost independent of bone‐specific ALP levels (*p* = 0.019). The incident rate of tooth loss among participants age 65 and older are 1.59 times the incident rate of younger participants. Estimates are from a Poisson model regressing number of teeth lost on bone‐specific ALP and age indicator. Excludes participants with premature tooth loss but unknown number (*N* = 3)

Dental defects including short roots and enlarged pulp chambers were rarely noted; two participants exhibited at least one of these features. 63% of participants had lost at least one permanent tooth prematurely, and 19% were missing eight or more teeth at the time of evaluation (Figure 2). Statistically significant associations were found between premature permanent tooth loss and ALP (*p* = 0.049) and between premature permanent tooth loss and bone‐specific ALP (*p* = 0.006) (Table 4). One of the novel distinct results of this study was that ALP level was associated with premature tooth loss; ALP level was negatively correlated (Spearman's *ρ* = −0.43) with number of teeth lost (i.e., the lower the ALP level, the more teeth lost). ALP level was also statistically significantly associated with number of teeth lost prematurely (*p* < 0.001) adjusting for age. The incident rate of tooth loss among patients age 65 years and over is 1.82 times the incident rate of younger participants' independent of ALP levels (Figure 3). Similarly, bone‐specific ALP was also associated with premature tooth loss; bone‐specific ALP level was negatively correlated (Spearman's *ρ* = −0.57) with number of teeth lost, and bone‐specific ALP was still associated with number of teeth lost prematurely (*p* < 0.001) adjusting for age (Figure 4).

**FIGURE 5:**
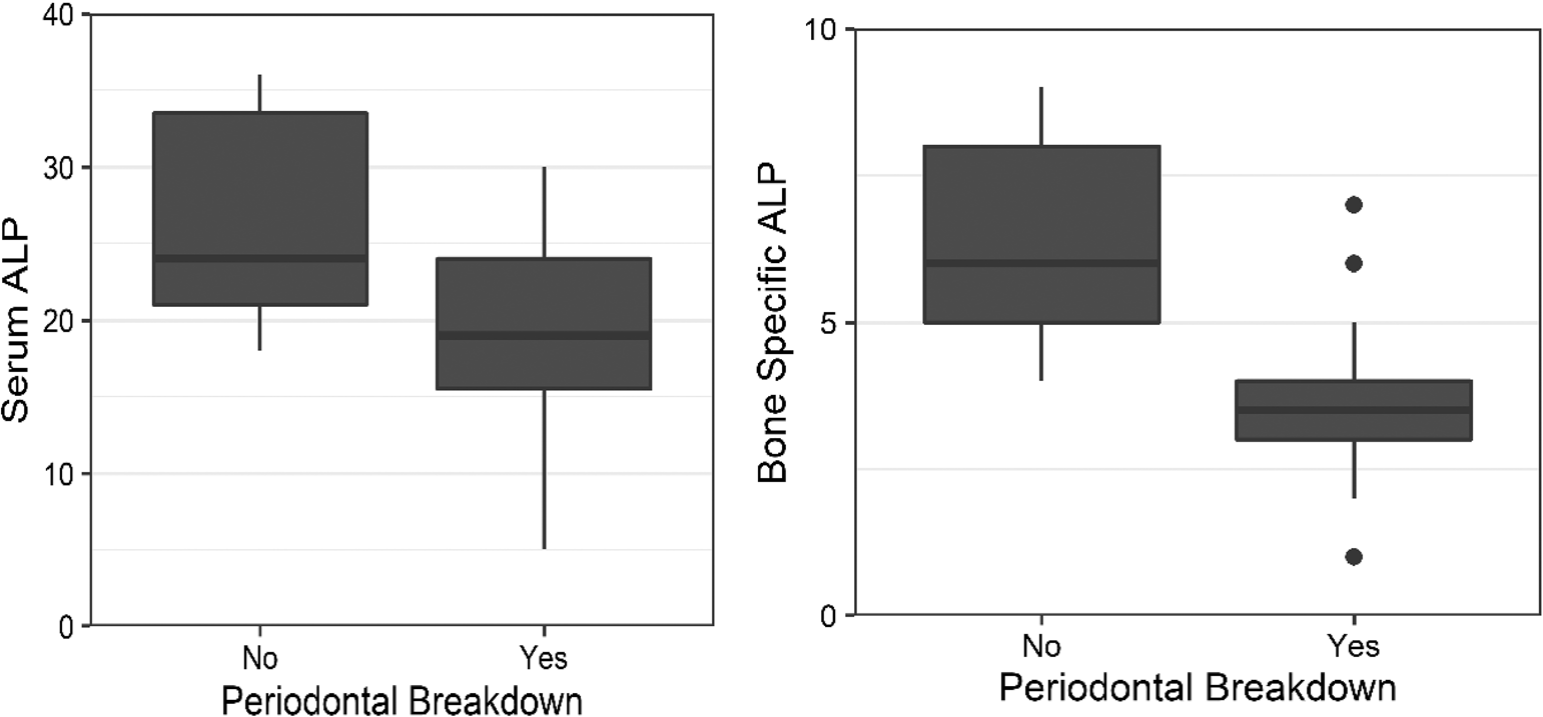
Participants with periodontal breakdown had significantly lower bone‐specific ALP (*p* = 0.014) than participants without periodontal breakdown and marginally lower serum ALP (*p* = 0.091)

All participants had periodontal disease, but the majority had early (or type II) periodontal disease. We found that neither ALP nor bone‐specific ALP were associated with periodontal disease status/severity (types I, II, III, IV, and VI) among this cohort. To try to overcome potential confounders of current periodontal status/severity, such as dental hygiene practices and past dental care, we separately analyzed periodontal breakdown. We then examined the association between current or historical periodontal breakdown with ALP levels. An inverse association of bone‐specific ALP levels by periodontal breakdown was statistically significant (*p* = 0.014); an inverse association between periodontal breakdown and ALP levels was marginally significant (Table 5 and Figure 5).

**TABLE 5:** Median ALP and bone‐specific ALP levels and IQR for Adult HPP participants by periodontal breakdown

	No periodontal breakdown	Periodontal breakdown	*p*‐value
	*N* = 6	*N* = 9	
Serum ALP	24.0 (12.5)	20.5 (8.5)	0.091
Bone‐specific ALP	6.0 (3.0)	4.0 (1.5)	0.014

Abbreviations: ALP, alkaline phosphatase.

Conversely, only a weak correlation was found between the coronal dental thickness and ALP levels (Spearman's *ρ* = −0.06). Dental caries was not associated with biomarkers, nor did we find that caries was associated with tooth loss.

## 4 DISCUSSION

There is a need for comprehensive evaluation of dental manifestations in adults with HPP. Increasing knowledge of dental manifestations may help improve identification of affected individuals who might otherwise escape diagnosis, guide dental management, and identify areas amenable to future research with the ultimate goal of improved patient health. Attempts to correlate the dental phenotype with disease biomarkers potentially allows prediction of dental phenotype. The results of this study confirm and expand upon previous research, as well as demonstrate novel findings. Dental manifestations such as caries, abscesses, enlarged pulp chambers, and short roots were uncommon in our unselected cohort of adult HPP, unlike findings described in previous studies.^20^ This difference could be partially explained because the majority of previous publications on dental findings in HPP were case reports, family studies, and small case series, and the majority of reports emphasized childhood HPP, adult survivors of childhood HPP, or odonto‐HPP. The current study was an unbiased, cross sectional evaluation of a cohort of adult individuals with HPP as opposed to reports of dental findings in HPP in patients who came to attention due to clinical signs or symptoms. A distinction must be made between loss of deciduous versus permanent teeth. Mori et al found that seven out of nine individuals with adult HPP experienced premature loss of deciduous teeth.^7^ However, among our study cohort, 67% recalled having no early loss of deciduous teeth. It is possible that a higher percentage experienced such loss but were too young to recall or had forgotten. Not unexpectedly, permanent tooth loss was common in adults with HPP in this unbiased cohort; 63% of participants had prematurely lost at least one permanent tooth, and 19% were missing eight or more teeth at the time of evaluation. Most participants recalled losing teeth due to either teeth becoming loose or cracked. However, these aspects were not explored in this study due to inability to measure tooth mineralization and incomplete historical information.

The etiology of tooth loss in HPP is unclear, but several interesting findings emerged. Tooth loss is generally primarily attributed to dento‐osseous changes underscored by alveolar bone loss secondary to defective mineralization, enamel hypomineralization, or cementum dysplasia. Although, it has been previously well described that premature tooth loss is a cardinal sign of HPP, prior studies had not examined the association between HPP biomarkers and tooth loss^6^ in an effort to begin to understand the etiology and predict the likelihood of its occurrence. We showed that ALP and bone‐specific ALP levels were both statistically significantly associated (*p* = 0.049 and *p* = 0.006 respectively) with premature permanent tooth loss in adults with HPP. The lower the biomarker levels, the higher the likelihood of tooth loss, suggesting that ALP, and especially bone‐specific ALP, play critical roles in tooth retention. As expected, there is more tooth loss in older participants age 65 years and over but nearly half of younger participants had also experienced premature tooth loss including two younger participants who had lost more than eight teeth.

Additionally, we explored the prevalence of periodontal disease in adults with HPP, its potential role in tooth loss, and association with biomarkers. Periodontal disease was common in the cohort of participants, moderate or severe periodontitis was observed in nearly 40%. Comparatively, in the general population approximately 11% of adults age 65 years and over manifest moderate or severe periodontal disease.^28^ In the study, 12 out of 27 were age 65 or older, indicating a significantly higher prevalence of periodontitis with ALP deficiency than the general population. Neither ALP nor bone‐specific ALP levels were associated with periodontal disease status/severity (types I, II, III, IV, VI) alone among this cohort. To try to overcome potential confounders affecting an association such as dental hygiene and care (generally good in this cohort) or treatment, we separately defined (see Methods) and analyzed periodontal breakdown. We then examined the association between current or historical periodontal breakdown with biomarkers. The inverse association between periodontal breakdown and bone‐specific ALP levels was statistically significant among participants of all ages. The lower the biomarker levels, the worse the periodontal breakdown. This finding suggests the need for additional research to determine if low bone‐specific ALP is also a risk factor for periodontal disease in individuals who do not have HPP. This may shed additional light on causes of periodontal disease in general. Although analyses were stratified by age to account for the effect of natural aging on periodontal disease and tooth loss, because there was only one participant without periodontal breakdown among participants age 65 years and over no statistical comparison could be made.

Limitations to the study are noted. Periodontal disease occurring solely or predominantly secondary to microbial activity may be difficult to distinguish from HPP (or alkaline phosphatase deficiency) and microbiological evaluation was not part of the study. A previous study has noted that HPP may produce favorable sites for colonization of periodontitis‐related bacteria resulting in periodontitis and alveolar bone loss and hence contributing to tooth loss.^29^ However, results of this study to a certain extent offset this limitation, as a majority of the participants in this cohort had good dental hygiene and access to dental care, and therefore, it is less likely that microbial activity is the predominant reason for periodontal breakdown or alveolar bone loss. Additional research on whether microbial flora causing periodontal disease and alveolar bone loss differ in healthy individuals compared with adults with HPP is warranted.

These results support previous reports of the common observation of early loss of permanent teeth among individuals with adult HPP.^5^, ^6^, ^14^, ^21^ However, the associations between tooth loss and periodontal breakdown and HPP biomarkers is novel. Since we found that low ALP and bone‐specific ALP were correlated with tooth loss, we speculate that the ALP enzyme is important for retention of teeth. Precisely how the ALP enzyme participates in maintenance of dentition is not clear. Others have pointed out that with defective mineralization in HPP, the minerals required for proper tooth and bone hardness and strength are lacking.^30^, ^31^ It is also possible that tooth loss in adults with HPP could be due to reduced strength of alveolar bone or teeth. Future research is indicated to address the role of alkaline phosphatase in periodontal health and retention of teeth.

One of the strengths of this study is that these findings were gathered from unselected individuals, those identified by low ALP level rather than diagnosed HPP patients, so the findings represent a valid estimate of the scope, prevalence, and severity of dental manifestations in adults with HPP. However, a limitation of this study is that it was a cross sectional evaluation of individuals at a single time point; we did not follow the participants longitudinally from childhood to adulthood, nor were dental or medical records reviewed. More information on the course of disease could be gathered using a case control study design and longitudinal studies. Also, in this cohort, the majority of participants were relatively homogenous from a specific geographic area and received care in a single health care system, which could introduce bias.

In conclusion, dental professionals and other providers should consider HPP in the differential diagnosis among individuals in whom unexpected premature tooth loss occurs. Since dental providers are those most likely to notice premature tooth loss, they can serve a critical role in early recognition and referral of HPP. These findings have implications for strategies or treatment modalities to prevent tooth loss in this cohort. Although it may be expected that early and aggressive prevention and/or treatment of periodontal disease can prevent tooth loss, this may not be sufficient, since in this study cohort, participants with good dental hygiene also lost teeth prematurely. To date, very few reports have investigated the dental parameters to determine the efficacy of ERT to prevent and/or reverse HPP‐associated dental‐periodontal defects centering exclusively on pediatric patients. According to recent case reports, early initiation of ERT resulted in stable periodontal conditions in deciduous teeth (teeth that erupted after initiation of ERT) due to improvement in alveolar bone and tooth mineralization.^18^, ^23^ ALP deficiency was associated with tooth loss. Thus, there is hope that ERT may help preserve dentition in HPP. Conducting future clinical investigation on the effect of ERT or other future treatments on dental manifestations may prove important in determining whether early treatment can prevent lifelong dental health problems and improve the quality of life for adults with significant dental manifestations of HPP.

## FUNDING INFORMATION

This study was sponsored by Alexion Pharmaceuticals, Inc. as an Investigator Initiated Study. The sponsor was provided the opportunity to review the manuscript as a courtesy prior to submission for publication, but had no role in study design, data collection and analysis, decision to publish, or preparation of the manuscript.

## COMPLIANCE WITH ETHICS GUIDELINES CONFLICT OF INTEREST

Robert D. Steiner reports equity interest in and consulting fees from Acer Therapeutics and PTC Therapeutics, research funded by Alexion and the Smith Lemli Opitz Syndrome Foundation. In the past 5 years, travel support from Pfizer to review clinical trial results. In the past 5 years consulting fees from Aeglea, Alexion, Best Doctors, Biomarin, E‐Scape Bio, Health Advances, Leadiant, Precision for Value, Retrophin/Travere, and Honoraria from Medscape/WebMD and The France Foundation. Robert Steiner is also an employee of PreventionGenetics. No additional potential conflicts of interest exist on the part of Dr. Steiner.

Priya Sinha, Rachel Gabor, Rachael Haupt‐Harrington, and Leila Deering declare no real or potential conflict of interest.

## ETHICS STATEMENT

This study was approved by the Marshfield Clinic Institutional Review Board. All procedures followed were in accordance with the ethical standards of the responsible committee on human experimentation (institutional and national) and with the Helsinki Declaration of 1975, as revised in 2000 (5). Informed consent was obtained from all patients for being included in the study. All participants consented to enrollment in the study.

## Data Availability

De‐identified data is available from the authors upon request.
